# Gene expression analysis of conjunctival epithelium of patients with Stevens-Johnson syndrome in the chronic stage

**DOI:** 10.1136/bmjophth-2018-000254

**Published:** 2019-06-16

**Authors:** Mayumi Ueta, Chie Sotozono, Hiromi Nishigaki, Suzuko Ohsako, Norihiko Yokoi, Katsura Mizushima, Yuji Naito, Shigeru Kinoshita

**Affiliations:** 1 Department of Frontier Medical Science and Technology for Ophthalmology, Kyoto Prefectural University of Medicine, Kyoto, Japan; 2 Department of Ophthalmology, Kyoto Prefectural University of Medicine, Kyoto, Japan; 3 Departmentof Molecular Gastroenterology and Hepatology, Kyoto Prefectural University of Medicine, Kyoto, Japan

**Keywords:** Stevens-Johnson syndrome, human conjunctival epithelium, gene expression analysis

## Abstract

**Objective:**

To investigate the pathology underlying the ocular surface complications of patients with Stevens-Johnson syndrome (SJS) in the chronic stage.

**Methods and analysis:**

Using oligonucleotide microarrays, we performed comprehensive gene expression analysis of the conjunctival epithelium of patients with SJS in the chronic stage (n=3). The controls were patients with conjunctival chalasis (n=3). We confirmed the downregulation and upregulation of transcripts of interest by quantitative real-time PCR (RT-PCR) assay. The expression of ocular surface protein with significantly upregulated transcripts was assessed immunohistochemically.

**Results:**

Compared with the controls, in the conjunctival epithelium of patients with SJS, 50 transcripts were downregulated by less than one-tenth (analysis of variance (ANOVA) p<0.05). Transcripts MUC7, PIGR, HEPACAM2, ADH1C and SMR3A were downregulated by less than one-fiftieth. 65 transcripts were upregulated more than 10- fold; the difference between patients with SJS and the controls was significant (ANOVA p<0.05). There were 14 transcripts that were upregulated more than 50-fold; they were SERPINB4, KRT1, KRTDAP, S100A7, SBSN, KLK6, SERPINB12, PNLIPRP3, CASP14, ODZ2, CA2, CRCT1, CWH43 and FLG. Quantitative RT-PCR of conjunctival epithelium samples from 11 patients with SJS and 26 controls showed that the gene expression of PIGR, HEPACAM2 and ADH1C was significantly downregulated while the gene expression of ODZ2 (teneurin-2) was significantly upregulated in patients with SJS. We document that teneurin-2 protein can be expressed in human conjunctival epithelium.

**Conclusion:**

Our results suggest that the downregulation of PIGR, HEPACAM2 and ADH1C and upregulation of teneurin-2 expression contribute to the pathology of the ocular surface in patients with SJS in the chronic stage.

**Key messages**

**What is already known about this subject?**

- The pathobiology of ocular surface complications in Stevens-Johnson syndrome (SJS) with severe ocular complications has been not well known, so we performed comprehensive gene expression analysis of the conjunctival epithelium, to further investigate about it.

**What are the new findings?**

- We found that the downregulation of PIGR, HEPACAM2 and ADH1C and upregulation of teneurin-2 expression contribute to the pathobiology of the ocular surface in patients with SJS in the chronic stage.

**How might these results change the focus of research or clinical practice?**

- Our present finding might contribute to resolve the mechanism of ocular complications of SJS.

## Introduction

Stevens-Johnson syndrome (SJS) is an acute inflammatory vesiculobullous reaction of the skin and mucosa such as the ocular surface, oral cavity and genitals. In patients with extensive skin detachment and a poor prognosis, the condition is called toxic epidermal necrolysis (TEN). Severe ocular complications (SOC) appear in about half of patients with SJS/TEN diagnosed by dermatologists.^1^ Cold medicines, including multi-ingredient cold medications and non-steroidal anti-inflammatory drugs (NSAID), were the main causative drugs of SJS/TEN with SOC in all patients with SJS and TEN. In fact, about 80% of our patients developed SJS/TEN with SOC after taking cold medicines a few to several days before disease onset.^2–5^ In the acute stage, patients with SJS/TEN with SOC manifest severe conjunctivitis with corneal and conjunctival erosion and a pseudomembrane in addition to skin eruption and erosion. Despite healing of the skin lesions, in the chronic stage of SJS/TEN with SOC, ocular surface inflammation persists^6^ as do ocular surface complications including conjunctival invasion into the cornea, symblepharon, ankyloblepharon and dry eye.^3 7^ It is not easy for ophthalmologists to render a differential diagnosis of SJS or TEN when patients present in the chronic stage because the vesiculobullous skin lesion expressed in the acute stage has healed by the chronic stage. Diagnosis of SJS/TEN in ophthalmology was based on a confirmed history of acute-onset high fever, serious mucocutaneous illness with skin eruptions and involvement of at least two mucosal sites including the ocular surface.^2 4 5 8–10^ Thus, ophthalmologists tend to report both SJS and TEN with SOC as ‘SJS’ in a broad sense.^3^

Conjunctival invasion into the cornea results in severe visual disturbance in patients with SJS with SOC in the chronic stage. Immunohistological differences in the characteristics of the conjunctival epithelium of patients with SJS with SOC and controls have been documented. We reported that EP3^11^ and EP4^12^ were markedly downregulated in the conjunctival epithelium of patients with SJS with SOC. To further investigate the pathology of ocular surface complications in SJS with SOC in the chronic stage, we performed comprehensive gene expression analysis of the conjunctival epithelium using GeneChip oligonucleotide microarrays (Affymetrix, Santa Clara, CA). We confirmed the downregulation and upregulation of transcripts of interest by quantitative real-time PCR (RT-PCR) assay and assessed the expression of protein with significant transcript upregulation by immunohistochemical methods.

## Materials and methods

### Patient and public involvement

This study was approved by the Institutional Review Board of Kyoto Prefectural University of Medicine. All experimental procedures were conducted in accordance with the tenets of the Declaration of Helsinki. Written informed consent was obtained from all patients after they were given a detailed explanation of the purpose of the research and the experimental protocols. Conjunctival tissue was obtained during ocular surface reconstruction or conjunctivochalasis surgery from the patients with their written informed consents, but patients did not involve in the recruitment to and conduct of the study. Our results will disseminate to study participants by paper and our web pages. There is no patient adviser in this study.

### Human conjunctival epithelium

For GeneChip analysis and quantitative RT-PCR, in vivo human conjunctival epithelium was harvested from conjunctival tissue obtained during ocular surface reconstruction or conjunctivochalasis surgery by overnight immersion at 4°C in 1.0 U/mL purified dispase (Roche Diagnostic, Basel, Switzerland).^13^

### Gene expression analysis

Gene expression profiles were investigated using a high-density oligonucleotide probe array (GeneChip, Human Gene 1.0 ST array (Affymetrix)). Total RNA was extracted with the Qiagen RNeasy Kit (Qiagen, Valencia, CA). We used approximately 764 885 probe sets covering more than 28 869 genes. Throughout the process we followed Affymetrix instructions. Scanned microarray images were obtained on a GeneChip Scanner 3000 7G (Affymetrix) using the default settings. Images were visually inspected to detect hybridisation artefacts.

### Quantitative RT-PCR

Total RNA was isolated using the RNeasy Mini Kit according to the manufacturer’s instructions. For the RT reaction we used ReverTra Ace (TOYOBO, Japan).

Quantitative RT-PCR assays were performed on a StepOnePlus instrument (Applied Biosystems) according to the manufacturer’s instructions. The primers and probes were purchased from Applied Biosystems. Quantification data were normalised to the expression of the housekeeping gene GAPDH.

### Immunohistochemistry

Human conjunctival tissue samples were from patients with SJS/TEN undergoing ocular surface reconstruction. The controls were nearly normal conjunctival tissue samples from operated patients with conjunctivochalasis.^11–14^

Conjunctival tissue sections mounted on slides were fixed for 10 min at 4°C with 4% PFA/0.1M phosphate-buffered saline (PBS), incubated overnight in a moist chamber at 4°C with sheep anti-human teneurin-2 polyclonal antibody (AF4578; R&D, MN, USA) or isotype control sheep IgG (R&D), and then washed in PBS without Ca and Mg [PBS(−)]. Alexa Fluor 488 donkey anti-sheep IgG (H+L) (Molecular Probes, Eugene, OR) was applied for 1 hour at room temperature. After washing the slides, antifade mounting medium with 4′,6-diamidino-2-phenylindole was applied (Vectashield; Vector Laboratories, Burlingame, CA).

### Data analysis

For microarray analysis we used the analysis of variance (ANOVA) p value to record significant differences between patients with SJS and the controls. Data from quantitative RT-PCR assays were expressed as the mean±SE and evaluated by the Student’s t-test using Microsoft Excel.

## Results

### GeneChip analysis: comparison of transcripts downregulated by less than one-tenth and upregulated more than 50-fold in conjunctival epithelium from patients with SJS and the controls

We subjected conjunctival epithelium from three patients with SJS with SOC and three patients with conjunctival chalasis to gene expression analysis by microarray. We found that 49 transcripts were downregulated by less than one-tenth and that there was a significant difference between the patients with SJS and the controls (ANOVA p<0.05) (online supplementary table 1a). Transcripts downregulated by less than one-fiftieth were MUC7, PIGR, HEPACAM2, ADH1C and SMR3A (table 1a). As shown in online supplementary table 1b, 62 transcripts were upregulated more than 10-fold; there was a significant difference between samples from the patients with SJS and the controls (ANOVA p<0.05). The 14 transcripts upregulated more than 50-fold were SERPINB4, KRT1, KRTDAP, S100A7, SBSN, KLK6, SERPINB12, PNLIPRP3, CASP14, ODZ2, CA2, CRCT1, CWH43 and FLG (table 1b); 6 of these 14 transcripts were related to cell differentiation or epidermis development in biological aspect.

10.1136/bmjophth-2018-000254.supp1 Supplementary data

**Table 1:** Transcripts regulated in the conjunctival epithelium of patients with SJS

Fold change	ANOVA P value*	Gene accession	Gene symbol	Gene description
Transcripts down-regulated by less than one -fiftieth in the conjunctival epithelium of patients with SJS				
−86.97	0.005	NM_001145006	*MUC7*	Mucin 7, secreted
−67.01	0.019	NM_002644	*PIGR*	Polymeric immunoglobulin receptor
−59.17	0.021	NM_001039372	*HEPACAM2*	HEPACAM family member 2
−53.46	0.019	NM_000669	*ADH1C*	Alcohol dehydrogenase 1C (class I), gamma polypeptide
−53.25	0.000	NM_012390	*SMR3A*	Submaxillary gland androgen regulated protein 3A
Transcripts upregulated more than 50-fold in the conjunctival epithelium of patients with SJS				
193.83	0.0023	NM_002974	*SERPINB4*	Serpin peptidase inhibitor, clade B, member 4
173.26	0.0001	NM_006121	*KRT1*	Keratin 1
150.7	0.0007	NM_207392	*KRTDAP*	Keratinocyte differentiation-associated protein
123.93	0.0007	NM_002963	*S100A7*	S100 calcium-binding protein A7
121.69	0.0019	NM_001166034	*SBSN*	Suprabasin
96.09	0.0046	NM_002774	*KLK6*	Kallikrein-related peptidase 6
91.02	0.0025	NM_080474	*SERPINB12*	Serpin peptidase inhibitor, clade B, member 12
89.35	0.0009	NM_001011709	*PNLIPRP3*	Pancreatic lipase-related protein 3
85.8	0.0008	NM_012114	*CASP14*	Caspase 14, apoptosis-related cysteine peptidase
68.67	0.0077	AK302302	*ODZ2*	Odz, odd Oz/ten-m homologue 2
53.44	0.0146	NM_000067	*CA2*	Carbonic anhydrase II
53.04	0.0188	NM_019060	*CRCT1*	Cysteine-rich C-terminal 1
52.76	0.0004	NM_025087	*CWH43*	Cell wall biogenesis 43 C-terminal homologue
51.27	0.0043	NM_002016	*FLG*	Filaggrin

*Difference from the control.

### Quantitative RT-PCR analysis: comparison of downregulated and upregulated transcripts in the conjunctival epithelium from patients with SJS and the controls

We subjected conjunctival epithelium from 11 patients with SJS and 26 patients with conjunctival chalasis to *quantitative RT-PCR assay* to confirm the less than one-fiftieth downregulation of five transcripts and the more than 50-fold upregulation of 14 transcripts.

**Figure 1:**
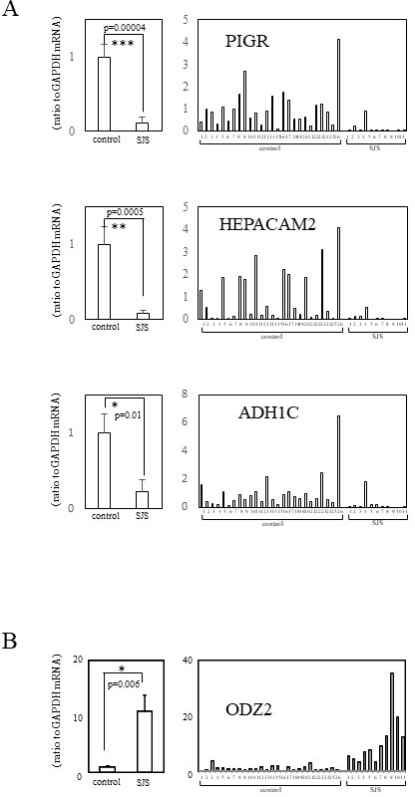
Quantitative real-time PCR (RT-PCR). (A) mRNA expression of the significantly downregulated genes in patients with SJS with severe ocular complications (SOC) compared with the control. (B) mRNA expression of significantly upregulated genes in patients with SJS with SOC compared with the control. Quantification data were normalised to the expression of the housekeeping gene GAPDH. The Y axis shows the increase in specific mRNA over the control samples. Data are the mean±SEM (controls, n=26; SJS, n=11). *p<0.05, **p<0.005, ***p<0.0005. SJS, Stevens-Johnson syndrome.

In patients with SJS, five transcripts tended to be downregulated; however, there was no significant difference between SJS and the controls with respect to MUC7 and SMR3A. Also, although 14 transcripts tended to be upregulated in SJS, there was no significant difference between patients with SJS and the controls with respect to 13 (SERPINB4, KRT1, KRTDAP, S100A7, SBSN, KLK6, SERPINB12, PNLIPRP3, CASP14, CA2, CRCT1, CWH43 and FLG). We attribute these findings to interindividual differences (online supplementary figures 1a,b). We concluded that the gene expression of PIGR (polymeric immunoglobulin receptor), HEPACAM2 (hepatic and glial cell adhesion molecule family member 2) and ADH1C (alcohol dehydrogenase 1C (class I), gamma polypeptide) was significantly downregulated and that ODZ2 (odd Oz/ten-m homologue 2), also called teneurin-2, was significantly upregulated in the conjunctival epithelium of patients with SJS with SOC (figure 1a,b).

10.1136/bmjophth-2018-000254.supp2 Supplementary data

### Protein expression of teneurin-2 in the conjunctival epithelium

**Figure 2:**
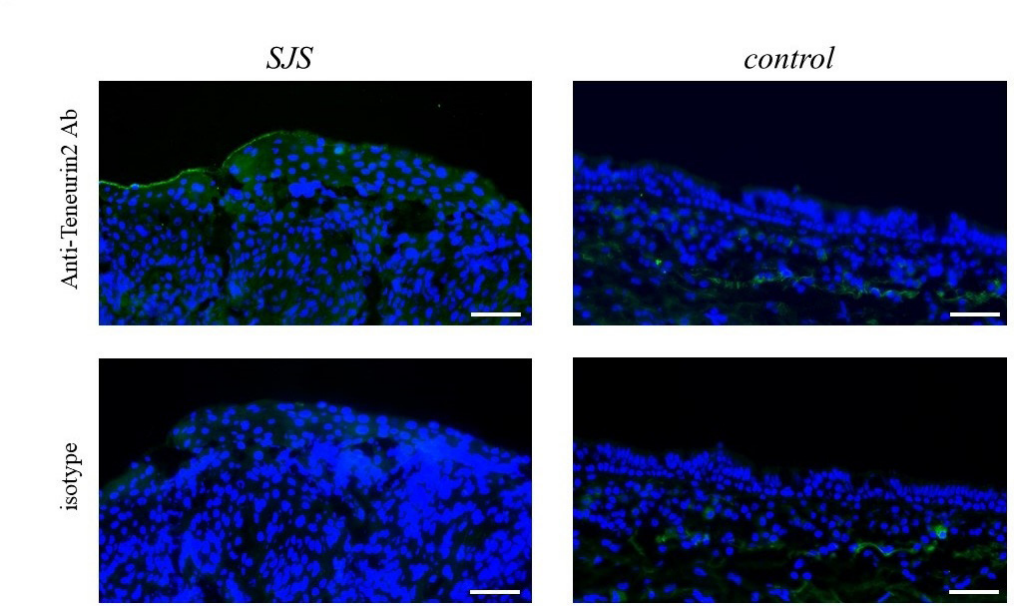
Teneurin-2 protein expression in conjunctival epithelium of patients with SJS with severe ocular complications (SOC). Immunohistochemistry detected teneurin-2 protein in the conjunctival epithelium of patients with Stevens-Johnson syndrome/toxic epidermal necrolysis (SJS/TEN). Bar: 50 µm.

We focused on the upregulated transcript, ‘teneurin-2’ and examined the protein expression. Our immunohistochemical studies revealed the expression of teneurin-2 protein on human conjunctival epithelium. Our findings suggest that despite individual differences, the degree of expression might be higher in patients with SJS with SOC than the controls (figure 2).

## Discussion

Our study of the pathology of ocular surface complications in patients with SJS with SOC in the chronic stage showed that in their conjunctival epithelium, the gene expression of PIGR, HEPACAM2 and ADH1C was significantly downregulated. The expression of ODZ2, also called teneurin-2, was significantly upregulated. We first document that teneurin-2 protein can be expressed in the human conjunctival epithelium.

PIGR, an epithelial glycoprotein that interacts with secretory IgA, is critical for regulating the secretory IgA level by transporting locally produced IgA.^15^ Because secretory IgA plays an important role in protecting mucosal surfaces such as the ocular surface against pathogens and antigens,^15^ the downregulated expression of the PIGR transcript in the conjunctival epithelium of patients with SJS with SOC may compromise immune protection of the ocular surface. Elsewhere we postulated an association between a disordered innate immune response and SJS with SOC.^3^ This hypothesis was based on our observation of an association between the onset of SJS/TEN with SOC and microbial infections: many patients exhibited prodromata, including non-specific fever, coryza and sore throat, ailments that closely mimic upper respiratory tract infections of viral or mycoplasma origin that are commonly treated with antibiotics and NSAIDs.^2–5 8^ In addition, our patients with SJS presented with opportunistic infection of the ocular surface by bacteria, especially methicillin-resistant *Staphylococcus aureus* and *S. epidermis*; their rate of detection was higher on the ocular surface of patients with SJS with SOC than patients with other devastating ocular surface disorders.^3^ We posit that in patients with SJS with SOC, abnormalities in innate immunity are responsible for opportunistic bacterial infections of the ocular surface.^3^ Moreover, these patients presented with persistent inflammation of the ocular surface which harbours commensal bacteria.^3^ Consequently, we think that the downregulation of PIGR results in compromised immune protection of the ocular surface and the anomalous innate immunity seen in patients with SJS with SOC.

*HEPACAM2* mediates the cellular-extracellular matrix and cell-cell interactions. This mediation is critical for the formation and maintenance of the cellular architecture and for normal biological processes, including the regulation of cell adhesion, proliferation, apoptosis, migration and differentiation.^16^ However, the clinical importance of *HEPACAM2* and of *ADH1C*, which plays an important role in alcohol metabolism, on the ocular surface remains unclear.

Teneurin-2 is a member of the teneurin family, teneurin 1–4. Teneurins were initially described as ten-a and the pair-rule gene ten-m/odz in *Drosophila*. Teneurins belong to a novel class of signalling molecules that function both at the cell surface as type II transmembrane receptors and, after the release of the intracellular domain, as transcriptional regulators.^17^ The nuclear localisation of its intracellular domain has been observed in vitro in mammalian cells. The teneurin function appears to be required for a fundamentally important signalling mechanism conserved in invertebrates and vertebrates; it impacts many processes that rely on cell-cell contact throughout development.^17^ Although the clinical importance of teneurin-2 on the ocular surface remains unclear, we found that teneurin-2 protein is expressed in human conjunctival epithelium and that it may be upregulated in the conjunctival epithelium of patients with SJS. To the best of our knowledge, this is the first report of teneurin-2 expression on the human ocular surface.

In summary, we suggest that the downregulation of *PIGR*, *HEPACAM2* and *ADH1C*, and the upregulation of *teneurin-2* expression contribute to the pathology involving the ocular surface of patients with SJS in the chronic stage.

## References

[1] Sotozono C, Ueta M, Nakatani E, et al Predictive factors associated with acute ocular involvement in Stevens-Johnson syndrome and toxic epidermal necrolysis. Am J Ophthalmol 2015;160:e222:228–37. 10.1016/j.ajo.2015.05.002 25979679

[2] Ueta M, Sotozono C, Nakano M, et al Association between prostaglandin E receptor 3 polymorphisms and Stevens-Johnson syndrome identified by means of a genome-wide association study. J Allergy Clin Immunol 2010;126:e1210:1218–25. 10.1016/j.jaci.2010.08.007 20947153

[3] Ueta M, Kinoshita S Ocular surface inflammation is regulated by innate immunity. Prog Retin Eye Res 2012;31:551–75. 10.1016/j.preteyeres.2012.05.003 22728145

[4] Ueta M, Kaniwa N, Sotozono C, et al Independent strong association of HLA-A*02:06 and HLA-B*44:03 with cold medicine-related Stevens-Johnson syndrome with severe mucosal involvement. Sci Rep 2014;4 10.1038/srep04862 PMC5381277 24781922

[5] Ueta M, Sawai H, Sotozono C, et al IKZF1, a new susceptibility gene for cold medicine-related Stevens-Johnson syndrome/toxic epidermal necrolysis with severe mucosal involvement. J Allergy Clin Immunol 2015;135:e1517:1538–45. 10.1016/j.jaci.2014.12.1916 25672763

[6] Ueta M, Nishigaki H, Sotozono C, et al Downregulation of interferon-γ-induced protein 10 in the tears of patients with Stevens-Johnson syndrome with severe ocular complications in the chronic stage. BMJ Open Ophthalmol 2017;1:e000073 10.1136/bmjophth-2017-000073 PMC5721640 29354711

[7] Sotozono C, Ang LPK, Koizumi N, et al New grading system for the evaluation of chronic ocular manifestations in patients with Stevens-Johnson syndrome. Ophthalmology 2007;114:1294–302. 10.1016/j.ophtha.2006.10.029 17475335

[8] Ueta M, Sotozono C, Inatomi T, et al Toll-like receptor 3 gene polymorphisms in Japanese patients with Stevens-Johnson syndrome. Br J Ophthalmol 2007;91:962–5. 10.1136/bjo.2006.113449 17314152 PMC2266833

[9] Ueta M, Sotozono C, Tokunaga K, et al Strong association between HLA-A*0206 and Stevens-Johnson syndrome in the Japanese. Am J Ophthalmol 2007;143:367–8. 10.1016/j.ajo.2006.09.029 17258541

[10] Ueta M, Sawai H, Shingaki R, et al Genome-wide association study using the ethnicity-specific japonica array: identification of new susceptibility loci for cold medicine-related Stevens-Johnson syndrome with severe ocular complications. J Hum Genet 2017;62:485–9. 10.1038/jhg.2016.160 28100913

[11] Ueta M, Sotozono C, Yokoi N, et al Prostaglandin E receptor subtype EP3 expression in human conjunctival epithelium and its changes in various ocular surface disorders. PLoS One 2011;6:e25209 10.1371/journal.pone.0025209 21966456 PMC3178633

[12] Ueta M, Sotozono C, Yokoi N, et al Prostaglandin E receptor 4 expression in human conjunctival epithelium and its downregulation in devastating ocular surface inflammatory disorders. Arch Ophthalmol 2010;128:1369–71. 10.1001/archophthalmol.2010.232 20938012

[13] Ueta M, Hamuro J, Nishigaki H, et al Mucocutaneous inflammation in the Ikaros Family zinc finger 1-keratin 5-specific transgenic mice. Allergy 2018;73:395–404. 10.1111/all.13308 28914974

[14] Yamada K, Ueta M, Sotozono C, et al Upregulation of Toll-like receptor 5 expression in the conjunctival epithelium of various human ocular surface diseases. Br J Ophthalmol 2014;98:1116–9. 10.1136/bjophthalmol-2013-304645 24820048

[15] Johansen F-E, Kaetzel CS Regulation of the polymeric immunoglobulin receptor and IgA transport: new advances in environmental factors that stimulate pIgR expression and its role in mucosal immunity. Mucosal Immunol 2011;4:598–602. 10.1038/mi.2011.37 21956244 PMC3196803

[16] He Y, Wu X, Luo C, et al Functional significance of the hepaCAM gene in bladder cancer. BMC Cancer 2010;10 10.1186/1471-2407-10-83 PMC2845116 20205955

[17] Tucker RP, Chiquet-Ehrismann R Teneurins: a conserved family of transmembrane proteins involved in intercellular signaling during development. Dev Biol 2006;290:237–45. 10.1016/j.ydbio.2005.11.038 16406038

