# Supplementary material for: Gene expression analysis of conjunctival epithelium of patients with Stevens-Johnson syndrome in the chronic stage
Source: BMJ Open Ophthalmol. 2019 Jun 16;4(1):e000254. doi: 10.1136/bmjophth-2018-000254 (PMC6579564; doi:10.1136/bmjophth-2018-000254)
Supplement: Supplementary data [file bmjophth-2018-000254supp001.docx]

Supplemental Table 1a

The 49 transcripts down-regulated less than one-tenth and showed significant differences (ANOVA p-value < 0.05) in the conjunctival epithelium of SJS

| Fold Change | ANOVA  p-value | Gene Accession | Gene Symbol | Gene Description |
| --- | --- | --- | --- | --- |
| -86.97 | 0.005 | NM_001145006 | MUC7 | mucin 7, secreted |
| -67.01 | 0.019 | NM_002644 | PIGR | polymeric immunoglobulin receptor |
| -59.17 | 0.021 | NM_001039372 | HEPACAM2 | HEPACAM family member 2 |
| -53.46 | 0.019 | NM_000669 | ADH1C | alcohol dehydrogenase 1C (class I), gamma polypeptide |
| -53.25 | 0.000 | NM_012390 | SMR3A | submaxillary gland androgen regulated protein 3A |
| -45.90 | 0.015 | NM_021572 | ENPP5 | ectonucleotide pyrophosphatase/phosphodiesterase 5 (putative) |
| -42.57 | 0.049 | NM_000096 | CP | ceruloplasmin (ferroxidase) |
| -39.40 | 0.005 | NM_000064 | C3 | complement component 3 |
| -36.64 | 0.002 | NM_003225 | TFF1 | trefoil factor 1 |
| -35.67 | 0.049 | NM_180991 | SLCO4C1 | solute carrier organic anion transporter family, member 4C1 |
| -28.51 | 0.003 | NM_145740 | GSTA1 | glutathione S-transferase alpha 1 |
| -28.43 | 0.009 | NM_001010893 | SLC10A5 | solute carrier family 10 (sodium/bile acid cotransporter family), member 5 |
| -27.68 | 0.042 | NM_002354 | EPCAM | epithelial cell adhesion molecule |
| -26.96 | 0.020 | NM_003154 | STATH | statherin |
| -25.07 | 0.015 | NM_000300 | PLA2G2A | phospholipase A2, group IIA (platelets, synovial fluid) |
| -23.40 | 0.002 | NM_003679 | KMO | kynurenine 3-monooxygenase (kynurenine 3-hydroxylase) |
| -21.22 | 0.011 | NM_001098484 | SLC4A4 | solute carrier family 4, sodium bicarbonate cotransporter, member 4 |
| -20.41 | 0.008 | NM_012431 | SEMA3E | sema domain, immunoglobulin domain (Ig), short basic domain, secreted, (semaphorin) 3E |
| -18.73 | 0.040 | NM_002354 | EPCAM | epithelial cell adhesion molecule |
| -18.46 | 0.033 | NM_001201 | BMP3 | bone morphogenetic protein 3 |
| -17.53 | 0.021 | NM_005845 | ABCC4 | ATP-binding cassette, sub-family C (CFTR/MRP), member 4 |
| -17.52 | 0.020 | NM_004616 | TSPAN8 | tetraspanin 8 |
| -16.91 | 0.007 | NM_004440 | EPHA7 | EPH receptor A7 |
| -16.69 | 0.043 | NM_024626 | VTCN1 | V-set domain containing T cell activation inhibitor 1 |
| -16.03 | 0.009 | NM_025208 | PDGFD | platelet derived growth factor D |
| -16.00 | 0.007 | NM_006408 | AGR2 | anterior gradient homolog 2 (Xenopus laevis) |
| -15.41 | 0.013 | NM_002443 | MSMB | microseminoprotein, beta- |
| -14.97 | 0.019 | NM_130808 | CPNE4 | copine IV |
| -14.88 | 0.005 | NM_005019 | PDE1A | phosphodiesterase 1A, calmodulin-dependent |
| -14.18 | 0.003 | NM_004056 | CA8 | carbonic anhydrase VIII |
| -14.08 | 0.012 | NM_003944 | SELENBP1 | selenium binding protein 1 |
| -14.07 | 0.016 | NM_172316 | MEIS2 | Meis homeobox 2 |
| -13.65 | 0.024 | NM_004447 | EPS8 | epidermal growth factor receptor pathway substrate 8 |
| -12.66 | 0.006 | NM_006685 | SMR3B | submaxillary gland androgen regulated protein 3B |
| -12.52 | 0.007 | NM_006528 | TFPI2 | tissue factor pathway inhibitor 2 |
| -12.30 | 0.029 | NM_033266 | ERN2 | endoplasmic reticulum to nucleus signaling 2 |
| -12.10 | 0.019 | NM_001012993 | C9orf152 | chromosome 9 open reading frame 152 |
| -11.72 | 0.036 | NM_017709 | FAM46C | family with sequence similarity 46, member C |
| -11.51 | 0.012 | NM_005518 | HMGCS2 | 3-hydroxy-3-methylglutaryl-CoA synthase 2 (mitochondrial) |
| -11.51 | 0.013 | NR_026543 | C21orf88 | chromosome 21 open reading frame 88 |
| -11.32 | 0.017 | NM_001128310 | SPARCL1 | SPARC-like 1 (hevin) |
| -11.06 | 0.000 | NM_012306 | FAIM2 | Fas apoptotic inhibitory molecule 2 |
| -10.69 | 0.045 | NM_033495 | KLHL13 | kelch-like 13 (Drosophila) |
| -10.45 | 0.036 | NM_015678 | NBEA | neurobeachin |
| -10.43 | 0.032 | NM_018728 | MYO5C | myosin VC |
| -10.34 | 0.049 | NM_000187 | HGD | homogentisate 1,2-dioxygenase |
| -10.34 | 0.049 | NM_000187 | HGD | homogentisate 1,2-dioxygenase |
| -10.21 | 0.006 | NM_022569 | NDST4 | N-deacetylase/N-sulfotransferase (heparan glucosaminyl) 4 |
| -10.20 | 0.027 | NM_007288 | MME | membrane metallo-endopeptidase |
| -10.17 | 0.007 | NR_036521 | LOC100128252 | hypothetical LOC100128252 |

Supplemental Table 1b

The 62 transcripts up-regulated more than 10-fold and showed significant differences (ANOVA p-value < 0.05) in the conjunctival epithelium of SJS

| Fold change | ANOVA  p-value | Gene Accession | Gene Symbol | Gene Description |
| --- | --- | --- | --- | --- |
| 193.83 | 0.0023 | NM_002974 | SERPINB4 | serpin peptidase inhibitor, clade B, member 4 |
| 173.26 | 0.0001 | NM_006121 | KRT1 | keratin 1 |
| 150.7 | 0.0007 | NM_207392 | KRTDAP | keratinocyte differentiation-associated protein |
| 123.93 | 0.0007 | NM_002963 | S100A7 | S100 calcium binding protein A7 |
| 121.69 | 0.0019 | NM_001166034 | SBSN | suprabasin |
| 96.09 | 0.0046 | NM_002774 | KLK6 | kallikrein-related peptidase 6 |
| 91.02 | 0.0025 | NM_080474 | SERPINB12 | serpin peptidase inhibitor, clade B, member 12 |
| 89.35 | 0.0009 | NM_001011709 | PNLIPRP3 | pancreatic lipase-related protein 3 |
| 85.8 | 0.0008 | NM_012114 | CASP14 | caspase 14, apoptosis-related cysteine peptidase |
| 68.67 | 0.0077 | AK302302 | ODZ2 | odz, odd Oz/ten-m homolog 2 |
| 53.44 | 0.0146 | NM_000067 | CA2 | carbonic anhydrase II |
| 53.04 | 0.0188 | NM_019060 | CRCT1 | cysteine-rich C-terminal 1 |
| 52.76 | 0.0004 | NM_025087 | CWH43 | cell wall biogenesis 43 C-terminal homolog |
| 51.27 | 0.0043 | NM_002016 | FLG | filaggrin |
| 41.8 | 0.0010 | NM_176823 | S100A7A | S100 calcium binding protein A7A |
| 41.7 | 0.0183 | NM_032488 | CNFN | cornifelin |
| 38.26 | 0.0058 | NM_018004 | TMEM45A | transmembrane protein 45A |
| 35.82 | 0.0058 | NM_020299 | AKR1B10 | aldo-keto reductase family 1, member B10 |
| 32.67 | 0.0088 | NM_001110219 | GJB6 | gap junction protein, beta 6, 30kDa |
| 28.65 | 0.0299 | NM_006919 | SERPINB3 | serpin peptidase inhibitor, clade B, member 3 |
| 28.21 | 0.0062 | NM_001432 | EREG | epiregulin |
| 27.72 | 0.0143 | NM_058173 | MUCL1 | mucin-like 1 |
| 26.13 | 0.0020 | NM_152443 | RDH12 | retinol dehydrogenase 12 |
| 23.82 | 0.0026 | NM_207407 | TMPRSS11F | transmembrane protease, serine 11F |
| 23.64 | 0.0105 | NM_001870 | CPA3 | carboxypeptidase A3 (mast cell) |
| 23.26 | 0.0075 | NM_144505 | KLK8 | kallikrein-related peptidase 8 |
| 22.84 | 0.0167 | NM_016321 | RHCG | Rh family, C glycoprotein |
| 22.35 | 0.0048 | NM_173483 | CYP4F22 | cytochrome P450, family 4, subfamily F, polypeptide 22 |
| 22.09 | 0.0407 | NM_207373 | C10orf99 | chromosome 10 open reading frame 99 |
| 22.02 | 0.0076 | NM_001099287 | NIPAL4 | NIPA-like domain containing 4 |
| 21.18 | 0.0464 | NM_133492 | ACER1 | alkaline ceramidase 1 |
| 20.17 | 0.0006 | NM_001460 | FMO2 | flavin containing monooxygenase 2 |
| 19.65 | 0.0413 | NM_014058 | TMPRSS11E | transmembrane protease, serine 11E |
| 19.5 | 0.0484 | NM_000165 | GJA1 | gap junction protein, alpha 1, 43kDa |
| 19.31 | 0.0028 | NM_012427 | KLK5 | kallikrein-related peptidase 5 |
| 19.3 | 0.0397 | NM_014058 | TMPRSS11E | transmembrane protease, serine 11E |
| 18.65 | 0.0398 | NM_025261 | LY6G6C | lymphocyte antigen 6 complex, locus G6C |
| 18.62 | 0.0394 | NM_025261 | LY6G6C | lymphocyte antigen 6 complex, locus G6C |
| 18.43 | 0.0265 | NM_002426 | MMP12 | matrix metallopeptidase 12 |
| 18.4 | 0.0163 | NM_004425 | ECM1 | extracellular matrix protein 1 |
| 17.67 | 0.0100 | NM_002160 | TNC | tenascin C |
| 17.46 | 0.0024 | NM_005073 | SLC15A1 | solute carrier family 15, member 1 |
| 16.66 | 0.0132 | NM_001785 | CDA | cytidine deaminase |
| 16.61 | 0.0430 | NM_025261 | LY6G6C | lymphocyte antigen 6 complex, locus G6C |
| 16.47 | 0.0213 | NM_148897 | SDR9C7 | short chain dehydrogenase/reductase family 9C, member 7 |
| 16.39 | 0.0201 | NM_198965 | PTHLH | parathyroid hormone-like hormone |
| 16.14 | 0.0236 | NM_005398 | PPP1R3C | protein phosphatase 1, regulatory subunit 3C |
| 15.67 | 0.0064 | NM_005557 | KRT16 | keratin 16 |
| 14.99 | 0.0060 | NM_002421 | MMP1 | matrix metallopeptidase 1 |
| 13.95 | 0.0247 | NM_015596 | KLK13 | kallikrein-related peptidase 13 |
| 13.89 | 0.0202 | NM_001001548 | CD36 | CD36 molecule |
| 13.67 | 0.0007 | NM_197965 | SLC10A6 | solute carrier family 10, member 6 |
| 13.59 | 0.0025 | NM_000962 | PTGS1 | prostaglandin-endoperoxide synthase 1 |
| 13.49 | 0.0090 | NM_001085 | SERPINA3 | serpin peptidase inhibitor, clade A, member 3 |
| 13.43 | 0.0062 | NM_003247 | THBS2 | thrombospondin 2 |
| 13.3 | 0.0023 | NM_003914 | CCNA1 | cyclin A1 |
| 12.72 | 0.0160 | NM_013230 | CD24 | CD24 molecule |
| 12.13 | 0.0387 | NM_000853 | GSTT1 | glutathione S-transferase theta 1 |
| 11.49 | 0.0022 | NM_002307 | LGALS7 | lectin, galactoside-binding, soluble, 7 |
| 10.96 | 0.0024 | NM_002307 | LGALS7 | lectin, galactoside-binding, soluble, 7 |
| 10.81 | 0.0089 | NM_002192 | INHBA | inhibin, beta A |
| 10.68 | 0.0201 | NM_001172439 | ENDOU | endonuclease, polyU-specific |
| 10.64 | 0.0374 | NM_004744 | LRAT | lecithin retinol acyltransferase |
| 10.36 | 0.0491 | NM_024421 | DSC1 | desmocollin 1 |
| 10.32 | 0.0196 | NM_014375 | FETUB | fetuin B |
| 10.25 | 0.0014 | NM_173505 | ANKRD29 | ankyrin repeat domain 29 |
