# Supplementary figures and images for: Gene expression analysis of conjunctival epithelium of patients with Stevens-Johnson syndrome in the chronic stage
Source: BMJ Open Ophthalmol. 2019 Jun 16;4(1):e000254. doi: 10.1136/bmjophth-2018-000254 (PMC6579564; doi:10.1136/bmjophth-2018-000254)

Supplementary Figure 1a

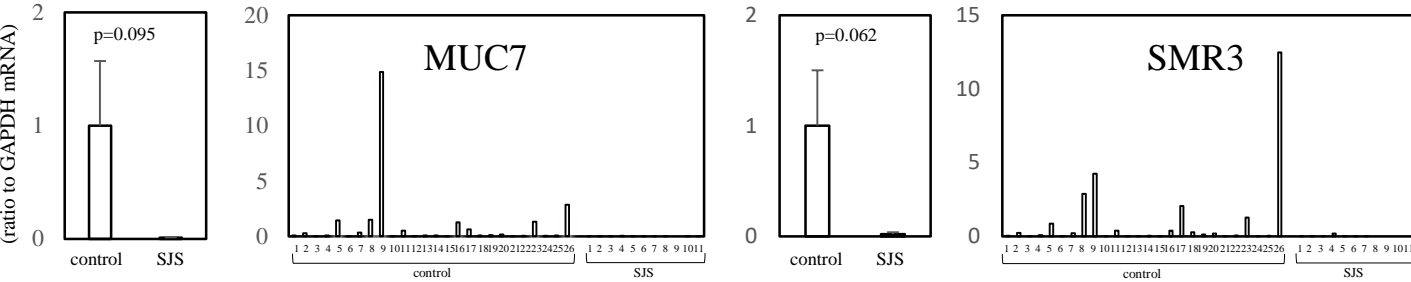

Supplementary Figure 1b

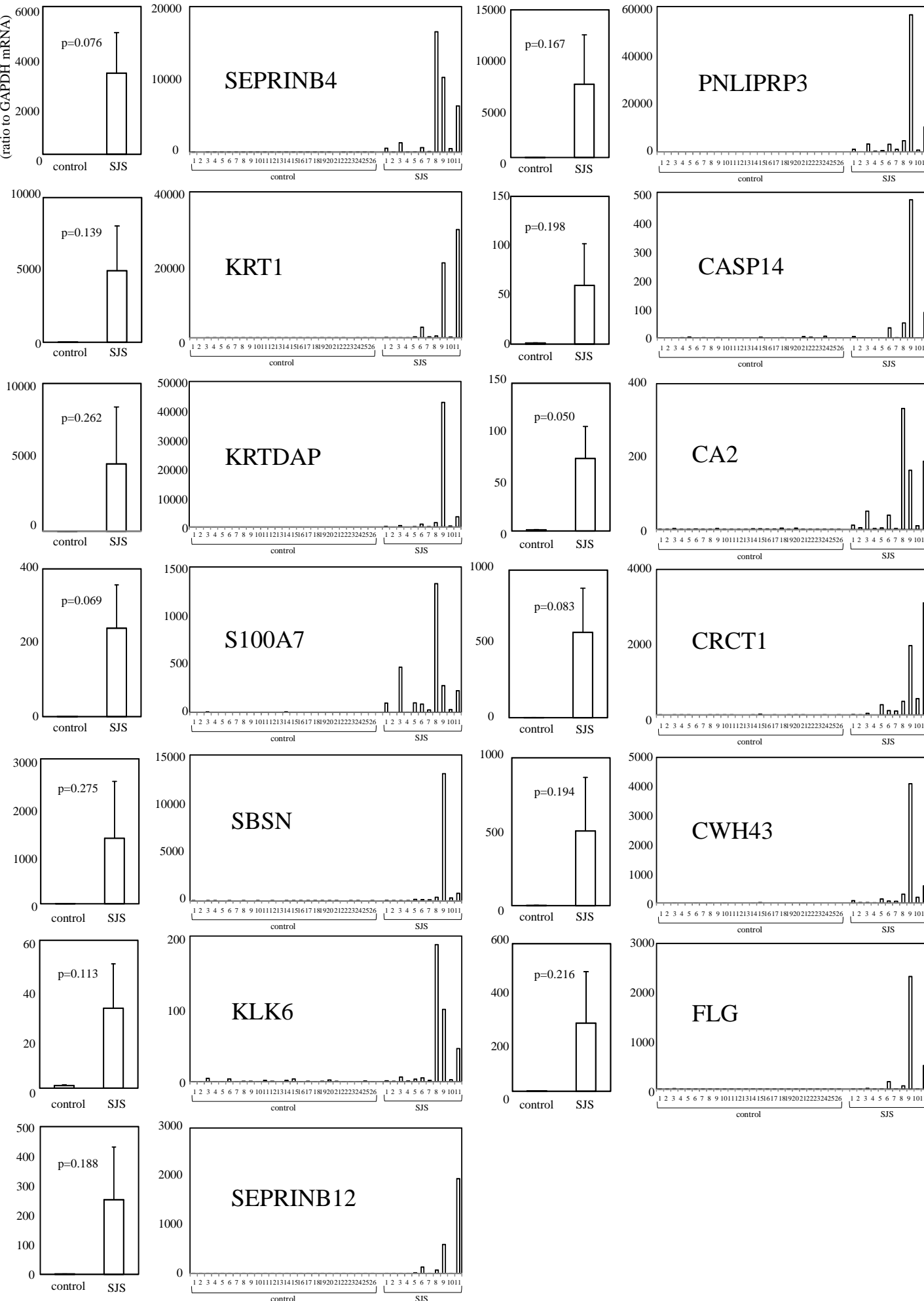

Supplement: Supplementary data [file bmjophth-2018-000254supp002.pdf]
